# Genetic and Molecular Regulation of Ecdysone Biosynthesis During Insect Metamorphosis

**DOI:** 10.3390/insects17080817

**Published:** 2026-08-06

**Authors:** Jie Zhang, Guanfeng Xu

**Affiliations:** College of Basic Medical Sciences, Zhejiang Chinese Medical University, Hangzhou 310053, China; zhangjiezcmu@126.com

**Keywords:** *Drosophila*, ecdysone, cholesterol, Halloween genes, transcription factors, chromatin

## Abstract

Steroid hormones govern diverse physiological processes during juvenile-to-adult development across vertebrates and invertebrates, ranging from pubertal initiation in mammals to metamorphosis in insects. Ecdysteroids, the collective name for many moulting hormones in insects, act as master effectors driving metamorphic development. Their biosynthesis begins with dietary cholesterol as the direct precursor (phytosterols are converted as needed) and is driven by a series of enzymatic reactions catalyzed by the evolutionarily conserved Halloween genes to produce ecdysone, which is ultimately converted into its potent metabolite 20-hydroxyecdysone (20E) in target tissues to induce extensive morphological and physiological changes. The precise temporal pattern of molting and metamorphosis is dictated by periodic ecdysone surges originating from the prothoracic gland. Given the extensive utility of *Drosophila* as a major model organism for investigating systemic growth and developmental timing, this review synthesizes recent insights into the regulatory mechanisms underlying ecdysone biosynthesis. In this review, we begin by outlining the ecdysteroid biosynthetic cascade and core catalytic enzymes in the prothoracic gland, followed by a systematic discussion of diverse regulatory strata, encompassing substrate availability, transcriptional regulation and chromatin accessibility, and their vital contributions to the control of ecdysteroid production.

## Simple Summary

Insects, like humans, rely on steroid hormones to control their growth and development. In insects, a hormone called ecdysone triggers the dramatic transformation from a larva into an adult, a process known as metamorphosis. This review explores how insect bodies precisely control the production of this crucial hormone. We focus on *Drosophila melanogaster*, a powerful model for studying these processes, and describe the complex “assembly line” of enzymes that manufacture ecdysone from dietary cholesterol. Importantly, we explain how this production is tightly regulated at multiple levels, from the availability of the raw material (cholesterol) to the expression of specific genes and the structural organization of DNA. Understanding this intricate control system is not only fundamental to biology but also has potential practical applications. For example, it could help to develop new, more targeted methods for controlling insect pests that damage crops or spread diseases by disrupting their hormone production without harming other non-insect organisms. Ultimately, this research elucidates the universal principles of hormone regulation, which are shared across the animal kingdom.

## 1. Introduction

Most animals undergo dramatic physiological and morphological changes throughout their life cycles. In mammals, puberty marks the transition from juvenile to sexually mature adulthood, while in insects, metamorphosis entails comprehensive structural remodeling across distinct developmental stages [1,2,3]. These developmental transitions are primarily driven by steroid hormones, a large family of cholesterol-derived compounds. In mammals, steroid hormones, including sex hormones and adrenal corticosteroids, play important roles in reproduction, metabolic homeostasis, and neurodevelopment [1,4,5]. In insects, the steroid hormones ecdysone and 20-hydroxyecdysone (20E) control metamorphosis, reproductive maturation, and diapause [6,7]. Despite the evolutionary distance, steroidogenesis in both mammals and insects exhibits remarkable conservation, with cholesterol serving as the common precursor and cytochrome P450 enzymes catalyzing key oxidative steps in the biosynthetic cascade [8,9,10]. Given this conservation, the relatively simple insect models, especially *Drosophila melanogaster*, which offers an unparalleled genetic toolkit [11], provides a powerful platform for dissecting the regulatory mechanisms of steroid biosynthesis. Such investigations not only deepen our understanding of insect development but also offer valuable insights into steroid hormone production in higher organisms.

**Figure 1:**
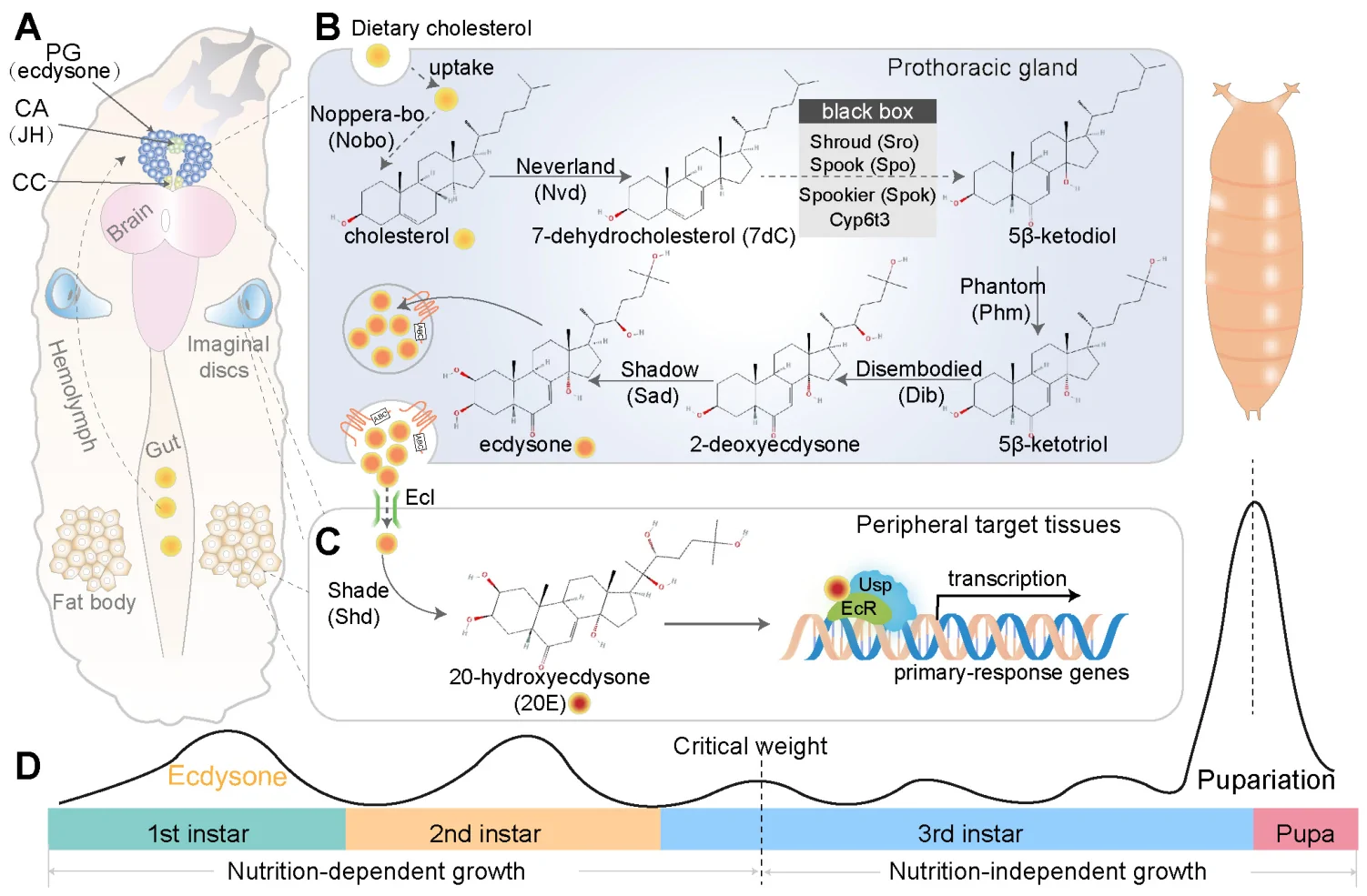
Ecdysone pulses coordinate the progression from larva to pupa in insects. (**A**) The left panel depicts the site of ecdysone synthesis in *Drosophila* larvae—the prothoracic gland (PG)—along with two of its target tissues, the imaginal discs and fat body. The PG (blue) is part of the ring gland, which also includes the corpus allatum (CA, green), which produces juvenile hormone, and the corpus cardiacum (CC, yellow); (**B**) The upper right panel illustrates the ecdysone biosynthetic pathway within PG cells, encompassing dietary cholesterol uptake, vesicular secretion, key intermediates, and the Halloween enzymes; (**C**) The lower right panel shows that ecdysone is secreted into the hemolymph in a pulsatile manner, transported into target tissues via the ecdysone importer (ECI), subsequently converted into its active form within target cells to trigger downstream signaling; (**D**) Of note, newly molted third-instar larvae trigger a small ecdysone pulse at critical weight (CW). Larvae starved prior to CW fail to undergo metamorphosis and die, whereas starvation after CW induces precocious metamorphosis.

During the metamorphic development in most insects, the prothoracic gland (PG) serves as the major endocrine organ responsible for converting dietary cholesterol into ecdysone, the immediate precursor of the active molting hormone 20E. In *Drosophila melanogaster*, the PG is a component of the composite ring gland, which also includes the corpus allatum and the corpora cardiaca [2,11] (Figure 1A). The genes encoding the key enzymes in the ecdysone biosynthetic pathway are collectively referred to as the Halloween genes, whose expression levels closely correlate with ecdysteroid titers in hemolymph. Following synthesis in the PG and secretion into the hemolymph via vesicular exocytosis, ecdysone is transported to peripheral target tissues such as the fat body and midgut via the ecdysone importer (EcI), where it is enzymatically converted into its biologically active form 20E [12,13,14,15]. Upon binding to the nuclear receptor complex, a heterodimer comprising the Ecdysone Receptor (EcR) and Ultraspiracle (Usp), 20E triggers a transcriptional cascade that orchestrates stage-specific developmental events [16] (Figure 1C). For example, 20E activates the AMPK-PP2A axis in the fat body to restrict growth rate [17], induces EcR.B2 expression in mature follicle cells to regulate ovulation [18], and affects wing size through TORC1 signaling [19].

The hemolymph 20E titer exhibits dynamic fluctuations during *Drosophila* metamorphosis, with each pulse triggering distinct developmental transitions, including larval molting and pupation [16] (Figure 1D). Both the synthesis and clearance of 20E are critical for orchestrating organismal development, underscoring the importance of precise steroidogenic regulation. Thus, elucidating the multi-layered and intricate mechanisms that govern ecdysone biosynthesis is vital for understanding developmental timing at the molecular level. In this review, we provide a concise overview of current research on ecdysone production and its regulatory networks. Following a brief summary of the ecdysone synthesis pathway in PG cells, we highlight recent advances in regulatory mechanisms of ecdysteroid biosynthesis at the levels of substrate availability, transcriptional control, and chromatin plasticity.

## 2. The Synthetic Pathway from Cholesterol to Ecdysone in PG Cells

In PG, the cholesterol serves as the substrate for ecdysone synthesis. However, since insects lack the capacity for de novo cholesterol biosynthesis, cholesterol acquisition is entirely dependent on dietary intake (Figure 1A). After ingestion, dietary cholesterol is loaded onto lipophorins and incorporated into lipid transfer particles, after which it is internalized into cells via receptor-mediated endocytosis [8,20,21]. Ecdysone biosynthesis is intimately coupled with cholesterol/steroid delivery, a process that relies on an autophagic cholesterol trafficking system involving Niemann–Pick complex 1 (Npc1) and the fatty acid elongase stuck in traffic (Sit) [3,22]. In *Drosophila*, the genes encoding the core enzymes of ecdysone biosynthesis are collectively termed Halloween genes, whose loss-of-function mutations lead to characteristic phenotypes, including embryonic lethality and abnormal, ghost-like cuticle structures. This gene family comprises multiple cytochrome P450 genes such as *Spook* (*Spo*), *Spookier* (*Spok*), *Cyp6t3*, *Phantom* (*Phm*), *Disembodied* (*Dib*), *Shadow* (*Sad*) and *Shade* (*Shd*), as well as the embryonic lethal genes *Neverland* (*Nvd*), *Shroud* (*Sro*) and *Noppera-bo* (*Nobo*) [6,12,23]. Briefly, the Rieske oxygenase Nvd initiates the biosynthetic cascade by catalyzing the dehydrogenation of cholesterol at the C-7 and C-8 positions to generate 7-dehydrocholesterol (7dC) [24]. Subsequently, 7dC is converted into the intermediate 5β-ketodiol via a series of reactions known as the “black box”, the detailed mechanisms of which remain incompletely elucidated. Nevertheless, several enzymes, including Sro [25], Spo/Spok [26] and Cyp6t3 [27], have been demonstrated to participate in this process. Following the “black box” reactions, 5β-ketodiol undergoes sequential hydroxylation at the C-25, C-22 and C-2 positions catalyzed by cytochrome P450 monooxygenases Phm [28], Dib [29] and Sad [30], ultimately yielding ecdysone (Figure 1B). The newly synthesized ecdysone is then secreted from the PG into the hemolymph via vesicle-mediated exocytosis and subsequently converted into its active form, 20E, through C-20 hydroxylation mediated by Shd, the only Halloween gene that is predominantly expressed in peripheral tissues rather than in the PG [13,31] (Figure 1C).

The core ecdysone biosynthetic pathway is highly conserved across species, although certain species-specific variations have been documented. Among the Halloween genes, *Nobo* is conserved only in dipteran and lepidopteran species and encodes a member of the glutathione S-transferase family. In *Drosophila*, loss-of-function mutants of *Nobo/GstE14* exhibit abnormal cholesterol accumulation in PG cells, and the resulting defects and developmental arrest can be almost completely rescued by exogenous cholesterol or 7dC. These findings suggest that *Nobo* plays a pivotal role in cholesterol transport and/or metabolism within the PG, rather than mediating the conversion of ecdysone intermediates [32] (Figure 1B). However, recent studies in the mosquito *Aedes aegypti* have demonstrated that Nobo has potent ketosteroid isomerase activity and may catalyze the double-bond isomerization of ketosteroids in the “black box” [33,34]. Although its precise biochemical function in vivo remains unclear, the essential role of Nobo in insect ecdysteroidogenesis is undisputed. Beyond *Nobo*, other Halloween genes also exhibit species-specific distribution patterns. For example, *Spok* and *Cyp6t3* are found only in *Drosophilidae* [26,32], whereas *Phm* is absent from the genomes of Myriapoda and Chelicerata, where ponasterone A, rather than 20E, serves as the major ecdysteroid [35]. Furthermore, in most lepidopteran insects, such as the *Bombyx* and *Manduca*, the PG primarily secretes 3-dehydroecdysone, which is subsequently processed into ecdysone in the hemolymph, and this is linked to their black box-specific product, 5β-diketol [2,36]. Collectively, the evolutionary conservation of Halloween genes confirms their core function in mediating ecdysone synthesis, while their species-specific divergence implies that different insects may have evolved alternative or branched biosynthetic pathways [37].

## 3. Cholesterol Availability Limits Ecdysone Biosynthesis at the Substrate Level

**Figure 2:**
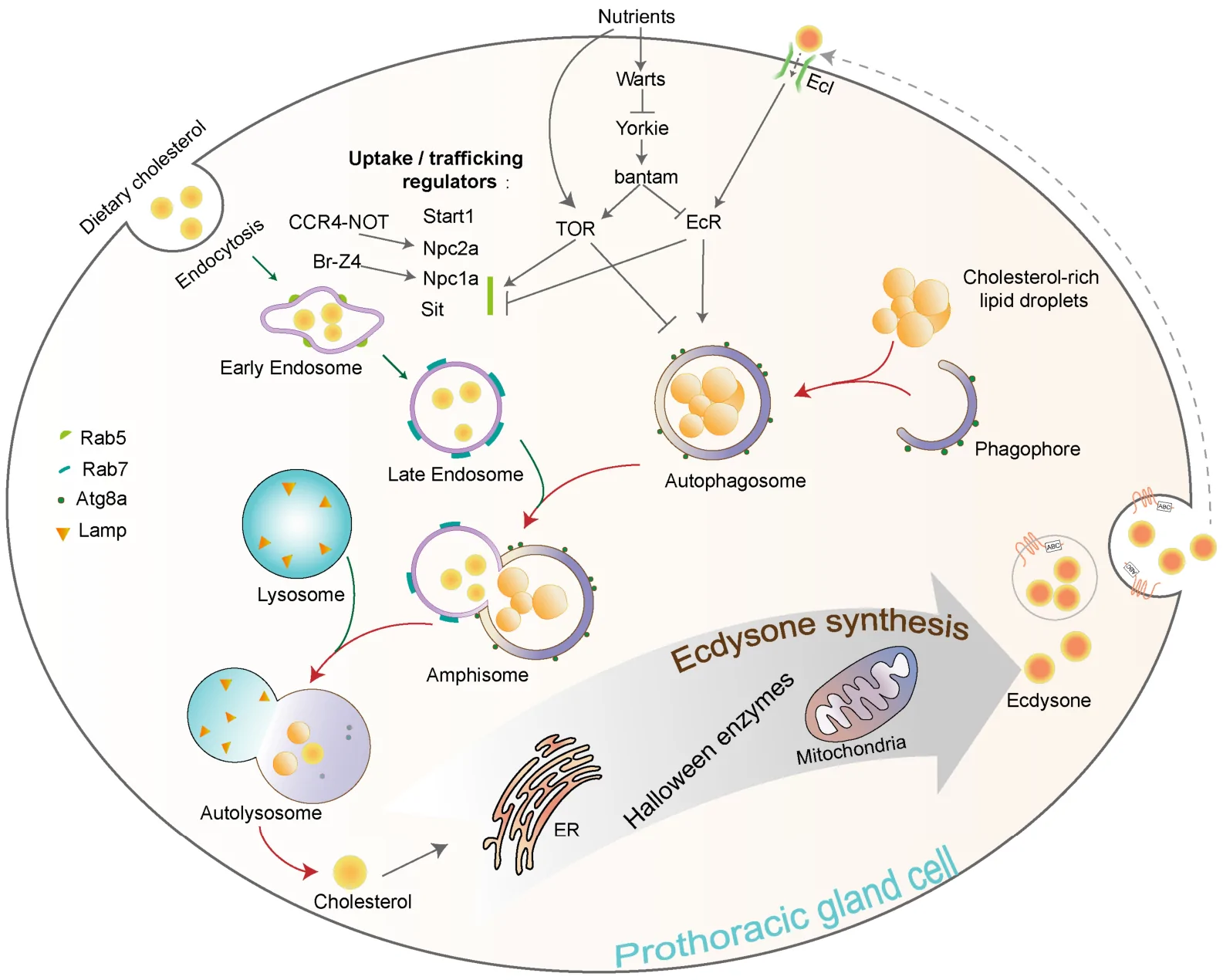
Autophagy-mediated cholesterol trafficking and substrate accessibility control during ecdysteroid biosynthesis in prothoracic gland cells. After cholesterol enters cells via receptor-mediated endocytosis, it is transported to lysosomes through the endo-lysosomal system. Within lysosomes, lipases hydrolyze cholesteryl esters to liberate free cholesterol, a process critically supported by autophagy-dependent cholesterol transport machinery. Autophagic vesicles sequester and transport cholesterol, as well as cholesterol-rich lipid droplets, and fuse with late endosomes and lysosomes to sequentially form amphisomes and autolysosomes, ultimately delivering cholesterol to the steroidogenic pathway. Cholesterol transport from the endo-lysosomal system to the ecdysone synthesis requires multiple protein players. Npc2a binds cholesterol and facilitates its transfer to the lysosomal membrane, whereas Npc1a exports cholesterol into the cytosol for steroidogenesis or storage within lipid droplets. Sit, a homolog of fatty acid elongases, participates in cholesterol trafficking and ecdysteroid production, while Start1 is proposed to mediate cholesterol delivery to mitochondria. Nutrient signals activate the Warts tumor-suppressor pathway, which through its downstream effector Yorkie regulates the microRNA *bantam*. *Bantam* in turn acts via downstream switches, TOR and EcR, whose mediated mechanisms modulate cholesterol uptake/trafficking as well as lipid mobilization and catabolism.

Following cholesterol entering PG cells, it is transported to the endoplasmic reticulum (ER) via the endolysosome system (indicated by the green arrow in Figure 2), where key biosynthetic enzymes, including Nvd, Spok, and Phm are localized. In the ER, cholesterol is converted into 7dC. Given the subcellular distribution of “black box”-related enzymes, the subsequent “black box” reactions also occur in this compartment [26,38]. The product of these reactions is then hydroxylated at the C-25 by Phm in the ER, after which the resulting intermediate is transported to the mitochondria, where the Dib and Sad are localized. After hydroxylation by these two enzymes in the mitochondria, the product is finally converted into ecdysone [2,3] (as shown in the gray-shaded area of Figure 2). The release of ecdysone from the PG into the hemolymph is mediated by calcium-stimulated, vesicle-dependent exocytosis, which requires the ABC transporter Atet to facilitate ecdysone transport from the cytoplasmic side to the luminal side of the vesicle membrane in an ATP-dependent manner [13]. In addition, specialized actin- and tubulin-based membrane protrusions, namely signaling filopodia, in the PG promote vesicle-mediated ecdysone secretion, and their developmental dynamics correlate with the establishment of ecdysone peaks [39].

The ecdysone biosynthetic system in *Drosophila* possesses remarkable substrate flexibility, utilizing a variety of phytosterols in addition to cholesterol. Although different sterol species can sustain development, they exert significantly differential regulation on the expression of 20E synthesis genes, all while engaging the same enzymatic machinery [40]. As dietary cholesterol-dependent organisms, insects must coordinate the integration of dietary cholesterol into ecdysone production through rigorous cholesterol trafficking and mobilization mechanisms. Several cholesterol transport proteins are critically involved in transporting cholesterol from the endo-lysosomal system to the steroidogenesis, including Niemann–Pick complex 1a (Npc1a), Niemann–Pick type C2a (Npc2a), and Start1. Both Npc1a and Npc2a promote the mobilization of intracellular cholesterol from the late endosome/lysosome system. Npc2 binds cholesterol and transfers it to the lysosomal membrane, whereas Npc1 inserts cholesterol into the lipid bilayer, after which cholesterol is transferred via membrane contact sites and vesicular transport to other cellular compartments [41]. Mutations in either gene result in abnormal sterol accumulation. However, *Npc1a* function is essential, as its loss leads to severe ecdysone deficiency and early lethality, whereas *Npc2a* mutants can survive to adulthood [42,43]. *Drosophila Start1* is highly expressed in PG and homologous to the vertebrate cholesterol transporter MLN64, suggesting that it may share a similar role in transporting cholesterol to mitochondria [44].

Additional regulators of cholesterol trafficking also play critical roles in ecdysone synthesis, such as stuck in traffic (Sit), Nobo, Carbon catabolite repressor 4-Negative on TATA (CCR4-NOT), and autophagy. Loss of *Sit*, which encodes a fatty acid elongase homolog, in the *Drosophila* PG leads to accumulation of free unesterified cholesterol, which is consistent with the *Npc1a*-deficient phenotype. Moreover, overexpression of *Sit* rescues the *Npc1a*-deficient phenotype, indicating that they share similar functions in cholesterol transport [22]. The *Drosophila* CCR4-NOT complex, a poly(A) degradation complex, is essential for cholesterol homeostasis in ecdysone synthesis and for maintaining normal nuclear structure of PG cells. Knockdown of its core component *Pop2* in the PG leads to downregulation of *Spok* and *Npc2* expression [45].

Autophagy, a conserved nutrient-regulated intracellular degradation process that sequesters cytoplasmic components into double-membrane vesicles (autophagosomes) for lysosomal recycling, also regulates the mobilization of cholesterol stored in lipid droplets within the PG, thereby ensuring timely steroid production [41] (indicated by the red arrow in Figure 2). In the *Drosophila* PG, Atg8a/LC3-positive vesicles have been observed to sequester cholesterol and fuse with lysosomes. Inhibiting autophagy by knocking out essential autophagy-related (*Atg*) genes leads to massive cholesterol accumulation in lipid droplets and reduced steroid production during late larval development, underscoring the importance of autophagy in delivering cholesterol for ecdysteroidogenesis [46,47].

Autophagic cholesterol trafficking in the *Drosophila* PG serves as a key hub connecting nutritional status to the timing of metamorphosis initiation. During the early third instar larval stage, larvae reach a nutritional restriction checkpoint termed the critical weight (CW), which represents the minimum body weight required for the larval-to-pupal transition. Upon passing this checkpoint, larvae rapidly produce sufficient 20E to trigger metamorphosis. Starvation before the CW blocks ecdysone production and causes developmental arrest, whereas starvation after the CW accelerates ecdysone synthesis and irreversibly induces premature metamorphosis [3,48] (Figure 1D). Nutritional signals regulate ecdysone production by controlling the autophagy-dependent mobilization of cholesterol from lipid droplets (LDs) into the biosynthetic pathway. The Hippo/Warts tumor-suppressor pathway mediates the effect of insulin on steroid signaling in *Drosophila* PG cells, thereby linking nutrient cues to steroidogenesis. Insulin/insulin-like growth factor signaling activates the Warts pathway, which in turn regulates the microRNA *bantam* through its downstream effector *Yorkie*. *Bantam* subsequently modulates autophagic cholesterol mobilization and substrate transport by regulating Target of rapamycin (TOR) and EcR signaling [47]. Before the CW, starvation-induced autophagy suppresses ecdysone production by interfering with cholesterol transport through interactions with late endosomes/lysosomes, thereby preventing developmental progression [49]. After the CW, however, starvation-activated autophagy promotes lipid droplet mobilization, accelerates ecdysone synthesis, and irreversibly induces premature metamorphosis [3]. These findings indicate that the autophagic cholesterol trafficking pathway serves as a key adaptive mechanism that ensures successful juvenile-to-adult transition under nutritional stress.

Interestingly, TOR signaling and EcR-mediated steroid feedback signaling in *Drosophila* PG jointly, yet antagonistically, regulate autophagic mobilization and cholesterol uptake/trafficking. During the feeding stage, TOR signaling is activated in response to nutrient intake and upregulates the expression of *Sit* and *Npc1a*, while inhibiting autophagy mobilization. In contrast, EcR acts as a negative regulator of *Sit* and *Npc1a*, downregulating their expression during non-feeding stages while promoting autophagy mobilization [22,47]. The expression of *Npc1a* in PG is also positively regulated by the ecdysone early-response gene Broad complex (*Br-C*) [50]. Br-C plays a crucial role in regulating the transcriptional program of numerous 20E-induced genes during insect metamorphosis. The *Drosophila* Br-C has four isoforms, denoted as Z1 to Z4, and all of which are expressed in the PG [51]. Notably, overexpression of the Z4 isoform, but not Z1–Z3, specifically upregulates *Npc1a* transcript levels [50].

In addition, the *Drosophila* hormone receptor 96 (DHR96) functions as a sterol sensor and occupies the top tier of the regulatory hierarchy controlling cholesterol homeostasis in insects. DHR96 responds to intracellular sterol availability and regulates sterol uptake, transport, and buffering capacity [8]. It binds dietary sterols and coordinates transcriptional responses to maintain cholesterol balance under both deficiency and excess conditions. Loss of *DHR96* renders flies hypersensitive to cholesterol deprivation and impairs their ability to tolerate high-cholesterol diets [8,52]. DHR96 is indispensable for mediating the transcriptional response to dietary cholesterol and acts as a key regulator of the *Npc* gene family, as well as of other genes involved in cholesterol uptake, metabolism, and transport [53]. Collectively, these factors and signaling cascades couple dietary cholesterol availability, cholesterol homeostasis, cholesterol uptake, trafficking and mobilization to systemic hormonal secretion (Figure 2).

## 4. Transcriptional Regulation of Ecdysone Biosynthesis

**Figure 3:**
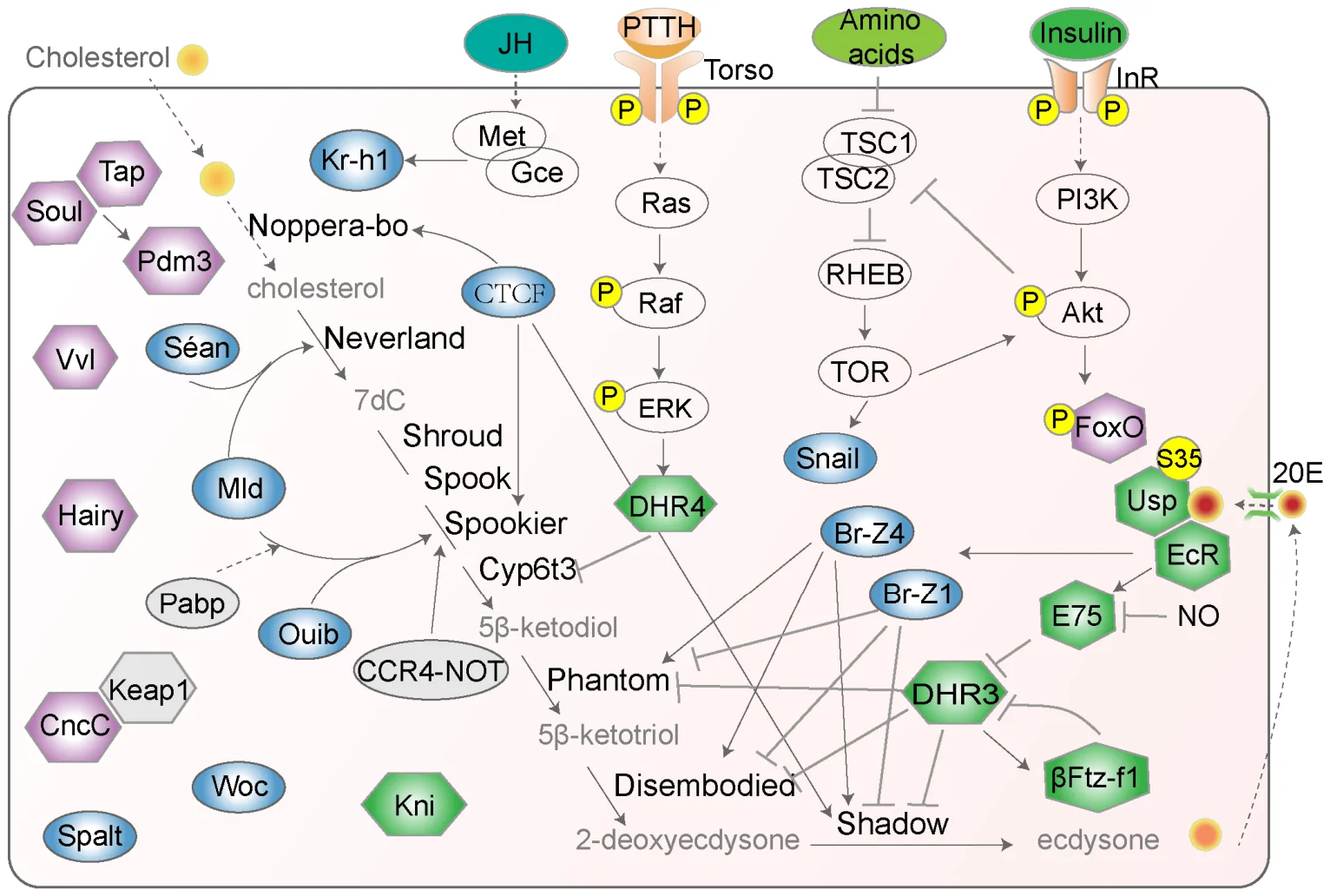
Overview of the biosynthetic pathway and transcriptional regulatory networks in the *Drosophila* PG. A large set of transcription factors (TFs) controls ecdysteroid biosynthesis by directly or indirectly modulating the expression of ecdysteroidogenic enzyme genes. Some signaling pathways may act on these TFs to regulate expression of the corresponding enzyme genes. For each signaling pathway, only key components are described. Among the TFs involved, C_2_H_2_ zinc-finger TFs are colored blue, nuclear receptors are shown in green, and other classes of transcription factors are depicted in purple. Yellow “P” labels indicate phosphorylation events.

The transcriptional levels of Halloween genes are closely correlated with the hemolymph ecdysone titer, and disruption of their expression leads to pupation delay or even death, underscoring the importance of tight transcriptional control during ecdysteroidogenesis. To date, many transcription factors (TFs) have been identified as direct or indirect regulators of Halloween gene expression. Moreover, various signaling pathways modulate the expression or subcellular localization of these TFs through cascaded regulatory events, ultimately affecting ecdysone synthesis [6,38]. Here, we briefly review the key TFs and related signaling pathways that govern ecdysone biosynthesis (Figure 3).

### 4.1. The Regulation by C_2_H_2_ Zinc Finger TFs

Numerous C_2_H_2_ zinc finger TFs have been shown to regulate the expression of single or multiple Halloween genes in the PG. Molting defective (Mld) is a zinc finger-associated domain (ZAD)-C_2_H_2_ zinc finger TF involved in ecdysone biosynthesis. Loss of *Mld* function in *Drosophila* PG specifically impairs *Nvd* and *Spok* expression without affecting other Halloween genes [54,55]. The lethal phenotype of *Mld* loss-of-function mutants can be fully rescued by simultaneous expression of *Nvd* and *Spo* [56]. Studies have further shown that poly(A) binding protein (Pabp) regulates *Spok* expression by modulating the nuclear localization of Mld. In *Pabp*-RNAi PG cells, Mld exhibits ectopic cytoplasmic accumulation, rather than the nuclear localization observed in wild-type cells. Notably, *Pabp*-RNAi does not alter the nuclear localization of other TFs [57]. Moreover, abnormal cytoplasmic aggregation of Mld is also observed in PG cells following knockdown of *Smt3*, a small ubiquitin-like modifier (SUMO) gene, which leads to reduced ecdysone titers and developmental arrest at the third instar [58]. Two additional ZAD-C_2_H_2_ zinc finger TFs, Séance (Séan) and Ouija board (Ouib), positively regulate the transcription of *Nvd* and *Spok*, respectively. Loss-of-function mutations in *Séan* or *Ouib* cause larval arrest, with significantly reduced expression of *Nvd* and *Spok*, respectively, without affecting other Halloween genes. These lethal phenotypes can be rescued by ectopic expression of *Nvd* or *Spo* alone [56,59]. Interestingly, Séan and Ouib function cooperatively with Mld to synergistically activate *Nvd* and *Spok* transcription. Co-expression of Séan and Mld in S2 cells induces more robust *Nvd* transcription than either factor alone. Similarly, co-expression of Ouib and Mld significantly induces endogenous *Spok* expression, and the response element for Séan-Mld on the *Nvd* promoter is nearly identical to that for Ouib on the *Spok* promoter [56]. However, among these three TFs, only *Ouib* is specifically expressed in *Drosophila* PG. Whether these ZAD-C_2_H_2_ TFs induce *Nvd* and *Spok* expression through PG-specific cofactors requires further investigation.

Other C_2_H_2_ zinc finger TFs, including CTCF, Krüppel-homolog 1 (Kr-h1), Snail, Without children (Woc), Spalt and Br-C, also exert essential regulatory functions. The insulator protein CTCF is a highly conserved zinc finger protein that plays critical roles in chromatin organization and gene expression during development in both *Drosophila* and mammals [60,61]. Loss of *CTCF* in *Drosophila* PG impairs the transcriptional activation of *Nobo*, *Spok* and *Sad*. The developmental delay caused by *CTCF* knockdown in the PG can be effectively rescued only by simultaneous feeding of 20E and cholesterol. Furthermore, *CTCF* knockdown in PG cells leads to lipid accumulation, indicating that CTCF is indispensable for Halloween gene expression and cholesterol homeostasis [62]. In insects, the sesquiterpenoid juvenile hormone (JH) acts as another key hormonal regulator alongside ecdysone, antagonizing ecdysone signaling to prevent precocious metamorphosis. Kr-h1 is a critical TF that mediates JH signaling. Specific knockdown of *Kr-h1* in the PG induces upregulation of *Nvd*, *Sro*, *Spok*, *Cyp6t3*, *Phm*, *Dib*, and *Sad* [63,64]. Further studies reveal that Kr-h1 binds directly to the *Spok* promoter to induce promoter DNA methylation, thereby repressing the transcription of steroidogenic enzymes [63].

The *Snail* gene family is evolutionarily conserved across metazoans [65,66]. *Drosophila Snail* is highly expressed in the larval PG. Both PG-specific overexpression and loss-of-function of *Snail* disrupt ecdysone biosynthesis and metamorphosis. PG-specific *Snail* RNAi leads to marked reductions in *Spok*, *Nvd*, *Phm*, *Sro*, *Sad*, and *Dib* levels, whereas PG-specific *Snail* overexpression significantly suppresses *Dib*, *Sro*, *Phm*, and *Sad* expression, indicating that *Snail* level must be tightly controlled in PG to ensure proper regulation of Halloween genes [67]. In *Drosophila* PG cells, endocycling mediated by the nutrient sensor TOR provides a necessary mechanism for the irreversible activation of metamorphosis. Inhibition of endocycling in the PG results in ecdysone deficiency and pupation defects [68]. The expression peak of *Snail* coincides with endocycling. Acting downstream of TOR, Snail serves as an endocycling regulator and functions as a molecular switch before and after the attainment of CW. Loss of *Snail* function causes endoreplication arrest before CW is attained, thereby preventing further larval development. However, after passing the CW checkpoint, *Snail* is no longer required for endoreplication or metamorphosis. Interestingly, the study also predicted that Snail binding sites in the promoters of *Sro*, *Phm*, *Dib*, and *Sad* (excluding *Nvd* and *Spok*) are conserved across several other *Drosophila* species, suggesting that these four Halloween genes may be directly regulated by Snail. Nevertheless, why both loss and overexpression of *Snail* reduce the transcription of Halloween genes requires further investigation [67]. The zing-finger protein Woc is required for ecdysone production, whose mutant larvae exhibit insufficient ecdysone synthesis and consequently fail to pupate [69]. Administration of 7dC, but not cholesterol, induces a sharp increase in ecdysone synthesis in *Woc* mutants and partially rescues development to the early pupal-adult stage [70]. However, no significant change in *Nvd* expression, which encodes the enzyme responsible for the conversion of cholesterol to 7dC, was detected in the *Woc* mutant larvae [71]. Given that Woc is a core component of the Heterochromatin Protein 1c (HP1c) transcription complex in *Drosophila*, which localizes to promoters of active genes and is required for transcription [72], the precise mechanism by which Woc regulates ecdysone synthesis is likely complex. The Spalt TFs—Spalt major (Salm) and Spalt-related (Salr)—are conserved nuclear proteins that contain several pairs of C_2_H_2_ zinc fingers and a glutamine-rich region [73]. The *Drosophila* PG requires the *Salm* and *Salr* functions for ecdysone synthesis, and their knockdown leads to larval developmental arrest. In *Salr*-knockdown PG, the expression of *Dib*, *Spok*, *Nvd*, and *Phm* is significantly reduced. Ectopic expression of *Phm*, but not *Nvd*, in the PG largely rescues the low pupation rate caused by *Salr* knockdown [74], indicating that part of Salr’s role in ecdysone synthesis may be mediated through regulating *Phm* expression.

Ecdysone can also regulate its own synthesis through positive and negative feedback on the PG. Positive feedback rapidly boosts ecdysone production to initiate pupation, whereas negative feedback represses ecdysone synthesis during the prepupal stage. These feedback loops rely on the expression of Br-C isoforms [75]. Br-C is an ecdysone-induced early protein that contains a protein–protein interaction BTB/POZ domain and a DNA-binding zinc finger domain. Its four isoforms (Z1-Z4) differ from one another in their C_2_H_2_ zinc finger sequences [76]. Br-Z4 appears at the late third instar, coinciding with the major high-titer ecdysone peak [51]. It mediates positive feedback regulation of ecdysone synthesis by binding directly to the promoters of *Phm* and *Sad* and activating their transcription, thereby promoting ecdysone production. In contrast, Br-Z1 drives negative feedback inhibition of ecdysone production by suppressing Halloween gene expression throughout the prepupal and pupal periods, which is critical for the decline in ecdysone titer during the prepupal phase. This Br-Z4/Z1-mediated feedback mechanism acts as a developmental switch to ensure rapid regulation of ecdysone synthesis in the PG, thereby precisely guiding the progression of metamorphosis [75]. In the antagonistic context between JH and 20E, Kr-h1 acts as a master TF that exerts dual regulatory control over *Br-C* expression. It represses *Br-C* during larval stages yet activates *Br-C* throughout pupation. Stage-specific SUMOylation of Kr-h1 serves as the molecular switch for this dual regulation. In early third-instar larvae, highly SUMOylated Kr-h1 interacts with the histone methyltransferase SmydA-8 to suppress *Br-C*; conversely, at the white prepupal stage, deSUMOylation disrupts the Kr-h1-SmydA-8 interaction, thereby turning on *Br-C* expression [77]. Although this study focused on the fat body, the JH-20E antagonism is systemic, and similar regulation may well operate in the PG as well.

### 4.2. The Regulation by Nuclear Receptors

In addition to the transcription factors discussed above, numerous nuclear receptors also contribute to the regulation of ecdysone biosynthesis, further increasing the complexity of this regulatory network. Both EcR and Usp, the heterodimeric partners that mediate 20E signal transduction in target tissues, are also involved in the control of ecdysone biosynthesis in the PG [75,78]. EcR is essential for Br-Z4/Z1-dependent ecdysone feedback control. In the *Drosophila* PG, inactivation of the ecdysone response via overexpression of a dominant-negative EcR represses the expression of Halloween genes, reduces ecdysone biosynthesis, and results in delayed pupation. By inducing distinct Br-C isoforms, EcR either activates or represses the transcription of ecdysone biosynthetic genes, thereby modulating both the rise and decline of ecdysone titers [75]. Usp fine-tunes ecdysone synthesis by interacting with the Forkhead box TF class O (FoxO). FoxO acts as a negative regulator of ecdysone synthesis in the *Drosophila* PG during the early third instar, when CW is reached and a small ecdysone peak triggers the developmental transition from larva to adult. Before CW, FoxO is localized in the nucleus of PG cells, where it directly binds to Usp to form a complex that suppresses the expression of *Phm* and *Dib*, thereby inhibiting ecdysone biosynthesis. After CW is reached, FoxO is exported from the nucleus into the cytoplasm, leading to a small 20E peak [78]. The attainment of nutrition-dependent CW is regulated by insulin/insulin-like growth factor signaling (IIS)/TOR signaling. Increased IIS/TOR activity in the PG leads to precocious ecdysone synthesis, premature CW attainment, and early metamorphosis [38]. Studies in *Drosophila* eye and imaginal discs have demonstrated that activated IIS/TOR signaling governs a cascade of phosphorylating kinases, including Akt, which in turn phosphorylates FoxO and triggers its nuclear export [79]. In the *Drosophila* PG, the nuclear localization of FoxO is also controlled by IIS/TOR activity. During the early third instar, reduced IIS/TOR activity in PG cells permits nuclear accumulation of the FoxO-Usp complex, which directly or indirectly restrains ecdysone biosynthesis. Upon larval feeding, upregulated IIS/TOR signaling induces FoxO phosphorylation, which drives phosphorylated FoxO out of the nucleus, dissociates the FoxO-Usp complex, and relieves the repression imposed on ecdysone synthesis [78].

Furthermore, the phosphorylation of the Usp itself can also affect its role in ecdysone synthesis. JH membrane signaling activates protein kinase C (PKC) via a putative receptor tyrosine kinase and the phospholipase C pathway. PKC subsequently phosphorylates Usp at Ser35, a site required for 20E to exert its maximal effects through its nuclear receptor complex EcR-Usp. In the *Usp*^S35A^ mutant, where Ser35 is replaced by Ala via genome editing, the mutant exhibits significantly reduced expression of *Phm*, *Dib*, and *Sad*, attenuated 20E signaling, and delayed developmental timing [80]. Binding of 20E to the EcR/Usp heterodimer induces the sequential expression of a cascade of early genes, including *E75*, *DHR3*, and *βFtz-f1*, which represent the 20E signaling cascade. These 20E-inducible nuclear receptors also participate in the autoregulation of ecdysone biosynthesis in PG cells [81,82]. Notably, βFtz-f1 is the first TF identified to modulate the expression of Halloween genes; loss of its function in *Drosophila* PG cells markedly reduces the protein levels of Phm and Dib [83]. However, subsequent studies revealed that βFtz-f1 does not directly activate the transcription of these enzymes, but rather acts by repressing the expression of *DHR3*, which functions as an inhibitory downstream component of the steroidogenic machinery [81]. Moreover, βFtz-f1 activity can be modulated by SUMOylation and acetylation. Knockdown of *Smt3* in PG cells causes a sharp decrease in βFtz-f1 abundance, whereas loss of the dATAC histone acetyltransferase complex results in elevated *βFtz-f1* mRNA levels but reduced protein levels [58,84]. DHR3 acts as a repressor of ecdysone production and downregulates the expression of steroidogenic enzymes at the onset of metamorphosis. Larvae in which *DHR3* is overexpressed in PG cells exhibit drastically reduced levels of Phm, Dib and Sad at the late third instar [81]. During this process, E75 positively regulates hormone biosynthesis [85]. In fact, E75 and βFtz-f1 indirectly activate the expression of Halloween genes by counteracting DHR3-mediated repression, thereby preventing premature suppression of ecdysone biosynthesis. Moreover, *E75* expression is regulated by EcR, while *DHR3* expression is regulated by E75 and βFtz-f1 [81]. Furthermore, nitric oxide (NO), as a short-range signaling molecule, can bind to the heme moiety of E75 and reverse its capacity to interfere with DHR3 [86].

Another 20E-responsive gene, *DHR4* [87], negatively regulates the biosynthesis of ecdysone, and the loss of its function within PG cells drives accelerated developmental progression. On the one hand, DHR4 suppresses expression of *Cyp6t3*, a gene that functions within the uncharacterized “black box” segment of the ecdysone biosynthetic pathway. On the other hand, DHR4 displays dynamic oscillation between the nuclear and cytoplasmic compartments of PG cells. When a low-titer ecdysone pulse occurs, DHR4 disappears from PG nuclei, and this nucleocytoplasmic trafficking is governed by the neuropeptide prothoracicotropic hormone (PTTH) [27]. PTTH is thought to control the timing of all major ecdysone peaks during insect development. In the *Drosophila* PG, PTTH activates the downstream Ras/Raf/ERK pathway through binding to its receptor Torso [88,89]. When PTTH or Torso function is abolished, this oscillatory behavior is blocked, leading to nuclear accumulation of DHR4. Conversely, hyperactivation of the PTTH pathway triggers translocation from the nucleus to the cytoplasm, which relieves DHR4-mediated repression of *Cyp6t3* and permits the generation of ecdysteroid pulses [27].

Knirps (Kni), a gap gene product, is critical for cell segmentation during early embryonic development [90]. Computational screening that covers the *Phm* promoter, *Dib* enhancer and *Spok* promoter has uncovered binding sites for Kni in all these regulatory regions. PG-specific knockdown of *Kni* downregulates the expression of *Phm*, *Dib*, and *Sad*, significantly reduces ecdysone titers, and causes developmental arrest at the first and second instar. Moreover, given that *Kni* expression is significantly upregulated in late third instar, inducible PG-specific silencing of *Kni* initiated at the second instar results in severe downregulation of nearly all Halloween genes, declined ecdysteroid production, and subsequent third-instar developmental arrest [55]. These findings demonstrate that Kni functions to turn on the expression of nearly all steroidogenic genes in PG cells at the late third instar.

### 4.3. The Regulation by Other Types of TFs

*Drosophila* cap-n-collar C (CncC) is a basic leucine zipper TF that interacts with its partner Keap1 (both are highly homologous to vertebrate Nrf2 and Keap1, respectively). In vertebrates, the Keap1-Nrf2 complex serves as a central regulator mediating transcriptional responses to exogenous stimuli and is closely associated with various human diseases. In *Drosophila*, reduction of either *CncC* or *Keap1* in PG leads to decreased ecdysone levels and delayed pupation. Loss of *CncC* function suppresses the transcription of *Nvd*, *Spok*, *Phm*, *Dib* and *Sad*, whereas depletion of *Keap1* only downregulates the expression of *Nvd*, *Spok* and *Phm*. Moreover, chromatin occupancy of both CncC and Keap1 has been detected at the loci of *Phm*, *Dib* and *Sad*, indicating that the two proteins directly govern ecdysteroid biosynthesis [91,92,93]. Similarly, studies in the ovary, another ecdysone-producing organ, have also demonstrated that loss of function of *CncC* or *Keap1* leads to reduced expression of several Halloween genes. And the C-terminal domain of Keap is crucial for its chromatin binding and the activation of ecdysone-biosynthetic gene expression [94]. In addition, loss of *CncC* in the PG also suppresses the precocious pupation induced by *Ras^V12^* expression and causes delayed pupation compared with wild-type larvae, suggesting that CncC acts downstream of the PTTH-Ras pathway [91].

The *Hairy* gene encodes a basic helix-loop-helix (bHLH) transcription factor that serves as a critical transducer within the JH repression cascade [95,96]. Hairy is capable of suppressing ecdysone biosynthesis. In *Drosophila*, PG-specific knockdown of *Hairy* upregulates the expression of Halloween genes (*Nvd*, *Spok*, *Phm*, *Dib*, and *Sad*), causing a rapid increase in hemolymph ecdysone titers and precocious larval–pupal transition. Conversely, overexpression of *Hairy* in the PG at the early third instar produces the opposite phenotypes, which can be partially rescued by 20E. Studies in the PG of *Bombyx* and *Drosophila* have shown that the polycomb repressive complex 2 (PRC2)-dependent histone H3K27 methylation mediates the repression of *Hairy* transcription, which is necessary for the ecdysteroid biosynthesis. During the final larval stage, H3K27me3 levels in the PG dynamically increase, and downregulation of PRC2 activity, leading to reduced H3K27me3, results in decreased ecdysone biosynthesis and disrupts the larval–pupal transition [97]. These studies provide insights into the antagonistic action between JH and ecdysone at the level of epigenetic regulation. Subsequent studies performed in the PG of final-instar *Drosophila* and *Bombyx* larvae reveal that histone deacetylase Rpd3-mediated H3K27 deacetylation induces transcriptional repression of *hairy* by promoting H3K27me3 accumulation at the *Hairy* locus, thereby facilitating ecdysone biosynthesis and the larval–pupal transition. Importantly, this switch from active H3K27 acetylation (H3K27ac) to repressive H3K27me3 at the *Hairy* locus is mediated by the homeodomain TF Schlank, which directly binds to the *Hairy* promoter and physically recruits Rpd3 and the PRC2 component Su(z)12. Moreover, the Schlank signaling cascade that represses *Hairy* transcription is inhibited by JH but positively regulated by ecdysone through a positive feedback loop [98].

Ventral veins lacking (Vvl), also known as Drifter (Dfr), is a member of the POU homeobox TF family, and its involvement in regulating ecdysone synthesis in the PG has been demonstrated in multiple species, including *Drosophila* [55], *Tribolium castaneum* [99], *Bombyx mori* [100], and *Oncopeltus fasciatus* [101]. PG-specific knockdown of *Vvl* in *Drosophila* downregulates the expression of *Sro*, *Spok*, *Phm*, *Dib*, and *Sad*, as well as ecdysone titers, resulting in developmental arrest at the first instar. Electrophoretic mobility shift assay (EMSA) has confirmed that Vvl binds to conserved sites in the *Phm* promoter, and given that Vvl-binding sites are also found in the *Dib* enhancer and the *Spok* promoter, these findings support that Vvl directly binds to promoters/enhancers to initiate transcription of ecdysone biosynthetic genes. Furthermore, *Vvl* is highly expressed in *Drosophila* PG cells, suggesting that Vvl may act as a conserved master regulator of steroidogenesis [55]. During standard protein synthesis, ribosomes continuously translate along the mRNA until reaching a stop codon, where release factors recognize the stop codon and mediate translation termination, with the normal error rate being less than 0.1% [102,103]. Translational stop codon readthrough (SCR) is considered an evolutionary strategy to increase the protein repertoire without genome expansion, allowing stop codons to be decoded as sense codons and producing protein isoforms with C-terminal extensions [104]. Studies have found that SCR of *Vvl* mRNA occurs at a rate of up to 50% in the *Drosophila* larval PG, and this C-terminal extension of Vvl is evolutionarily conserved in Diptera. Elimination of *Vvl* translational readthrough via CRISPR/Cas9-mediated gene editing reduces the expression of *Nvd*, *Spok*, *Dib* and *Sad*, inhibits 20E titer, and prolongs larval development as well as delays metamorphosis in the last instar. This indicates that the C-terminal extension of Vvl is essential for proper pupation by enhancing its ability to regulate target genes. Moreover, SCR of *Vvl* is controlled in a spatiotemporally regulated manner. The extended Vvl isoform works together with TF Mld to facilitate the formation of the transcription initiation complex and ensure appropriate activation of downstream target genes [103]. *Drosophila* Soul encodes a PG-specific bHLH TF that forms a heterodimer with another bHLH TF, Tap. Interference with either *Soul* or *Tap* causes strikingly similar phenotypes, including small and fragmented PG, elimination of almost all Halloween gene expression, larval arrest, and inability to undergo metamorphosis. More importantly, the expression peaks of both *Soul* and *Tap* in the PG occur before CW, and interfering with their function before (but not after) CW leads to third-instar larval lethality and disrupts the expression of a cohort of genes enriched in regulators and components of the steroidogenic biosynthetic pathway. Interestingly, a chitin-based cuticle gene (*Cpr49Ah*) and a TF gene (*pdm3*) are direct target genes of the Soul/Tap complex [105]. The *pdm3* encodes a PG-specific TF with a POU domain similar to that of Vvl [106]. PG-specific knockdown of *pdm3* results in downregulation of the Halloween gene expression as well as key transcription factor genes involved in ecdysone synthesis, including *Snail*, *Ouib*, *Séan*, and *Vvl*. Together, these results indicate that the Soul/Tap heterodimer is essential for PG development and the initiation of metamorphosis and functions at the top of the gene regulatory hierarchy in driving PG development, establishing CW, and producing steroid hormones [105].

## 5. Chromatin Accessibility in the Regulation of Ecdysone Biosynthesis

Beyond transcriptional regulators, chromatin state and chromatin regulators are also directly involved in regulating the expression of ecdysone biosynthetic genes. dATAC (ADA-Two-A-Containing) participates in the acetylation of lysine 5 and lysine 12 on histone H4 (H4K5ac and H4K12ac). The expression of Halloween genes, including *Spok*, *Phm*, *Dib*, and *Sad*, is downregulated in the PG of dATAC subunit mutants, demonstrating that the dATAC complex is indispensable for steroid biosynthesis [107]. Subsequent studies have revealed that beyond indirectly modulating the acetylation of *Ftz-f1* to alter its mRNA and protein abundance, the dATAC HAT complex may also directly regulate the transcription of Halloween genes by mediating H4K5 acetylation at their promoters [84].

Endoreplication in PG cells is thought to constrain ecdysteroid biosynthesis. Nutrient-dependent endoreplication functions as an intrinsic developmental timer in *Drosophila* PG cells. TOR-mediated PG endocycling is indispensable for triggering ecdysteroid biosynthesis, and blocking endocycling reduces ecdysone synthesis and causes larval developmental arrest [68]. Endocycling cells arise from mitotic cells via a cell-cycle switch termed the mitotic-to-endocycle switch (MES), which initiates cell growth and terminal differentiation. Downregulation of Fizzy-related (*Fzr*) in the PG impairs MES and subsequent ecdysone biosynthesis, whereas the chaperonin TCP-1 ring complex (TRiC) supports proper MES and endocycle progression by regulating Fzr folding [108]. The lysine demethylase 5 (KDM5) family proteins are chromatin-dependent transcriptional regulators encoded by a single gene in *Drosophila*, which catalyzes demethylation of trimethylated lysine 4 on histone H3 (H3K4me3). KDM5 is required for promoting endoreplicative cell cycles in PG cells and for transcription of ecdysone-synthetic enzymes. In *KDM5* mutant larvae, the expression of *Nvd*, *Spok*, and *Dib*, as well as 20E titers and endoreplication, are all significantly reduced, leading to developmental delay and pupal lethality. Restoring *KDM5* expression specifically in the PG is sufficient to rescue these phenotypes caused by *KDM5* null mutations. Mechanistically, KDM5 may activate the Torso/MAPK pathway to promote cell polyploidization, thereby facilitating ecdysone biosynthesis [109]. The CCR4-NOT complex is a highly conserved multi-protein assembly whose functions cover nearly all layers of gene expression regulation, with distinct tissue-specific roles. In *Drosophila* PG cells, CCR4-NOT executes nuclear functions. PG-specific knockdown of its core component *Pop2* leads to abnormal chromatin fragmentation and compaction, accompanied by a hollow sphere-like nucleolar morphology, as well as suppressed *Spok* expression. Collectively, these ultimately result in the arrest of larval development and the inability to initiate metamorphosis [45].

In the mature third-instar larval PG of *Drosophila*, Salm, one of the Spalt TFs (Salm and Salr), is highly enriched around the nucleolus, and they play a vital role in maintaining the nuclear genomic architecture. PG-specific knockdown of *Spalt* causes displacement of DNA toward the nuclear periphery, enlarged nucleoli, and loss of circular nuclear envelope morphology. Studies suggest that the impaired function of nuclear pore complexes in *Spalt*-knockdown PG cells compromises ERK phosphorylation and its nuclear localization, leading to reduced ERK signaling and subsequently decreased expression of *Phm*, *Spok*, *Dib*, and *Nvd* [74]. Importantly, *Nvd* and *Spok* are located in pericentric heterochromatin regions [56,77]. Genes residing in heterochromatin require the heterochromatin environment for normal expression [110,111]. It has been found that Su(var)2-10 and Su(var)205 regulate the transcription of the heterochromatic ecdysone biosynthetic gene *Nvd* in *Drosophila melanogaster* [112]. Both *Su(var)2-10* and *Su(var)205* belong to the suppressor of variegation [*Su(var)*] genes, which regulate the balance between euchromatin and heterochromatin in *Drosophila* and are essential for heterochromatin formation and the normal expression of heterochromatin-resident genes [113,114]. Knockdown of *Su(var)2-10* and *Su(var)205* in the PG reduces *Nvd* expression, leading to defects in the larval-to-prepupal transition. Given that Su(var)2-10 protein localizes near pericentric heterochromatin, it is proposed that the regulation of the inherent heterochromatin structure at the *Nvd* gene locus by Su(var)2-10 and Su(var)205 is critical for its transcriptional activation [112].

## 6. The Effects of Other Regulatory Factors

Glutathione (GSH), a bioactive tripeptide composed of glutamate, cysteine, and glycine, is essential for steroidogenesis. The *Drosophila* γ-glutamylcysteine synthetase catalytic subunit (*Gclc*) gene encodes the conserved catalytic enzyme that ligates glutamate and cysteine in GSH synthesis. Larvae lacking *Gclc* display severe GSH deficiency, reduced 20E titers, and developmental arrest at the second instar. However, 20E administration only allows *Gclc* mutant larvae to grow to the third instar, but has no significant effect on their subsequent development. Moreover, *Gclc* shares functions analogous to *Nobo* in cholesterol trafficking and/or metabolism, and its mutant larvae exhibit aberrant lipid accumulation within PG cells [115].

A series of cytochrome P450 enzymes encoded by Halloween genes are iron-containing proteins. Interfering with iron–sulfur cluster biogenesis or with the ferredoxins that supply electrons for steroidogenesis in the prothoracic gland impairs ecdysone synthesis and causes developmental arrest at the third instar [116,117]. *Drosophila* mitoferrin (*dmfrn*) is a mitochondrial carrier protein responsible for mitochondrial iron import. Under dietary iron deficiency or partial functional impairment of this ferritin, *dmfrn* mutant larvae fail to grow and initiate metamorphosis, and their developmental defects are partially attributed to insufficient ecdysteroid production. When *dmfrn* mutant larvae are raised under iron-replete conditions, the expression of *Dib* and *Sad* is markedly upregulated (likely a compensatory response to reduced enzymatic activity), accompanied by restored 20E synthesis [118]. These findings indicate that physiological mitochondrial iron supply is required for the biogenesis of iron–sulfur clusters and heme in insect PG, which is fundamentally essential for ecdysteroid biosynthesis. In ecdysone biosynthesis, Dib and Sad, the enzymes that catalyze the last two hydroxylation steps, are mitochondrial cytochrome P450s [30]. Dib was predominantly localized to the mitochondria under well-fed conditions, as observed using a Dib-mCherry knock-in reporter. However, its mitochondrial localization undergoes dynamic remodeling upon starvation before and after CW. Before CW, prolonged starvation progressively impairs the import of Dib into mitochondria. Moreover, inhibition of PTTH signaling, insulin signaling, or TOR activity prior to CW all reduces mitochondrial import of the Dib protein [3]. Combined with the reduced ecdysteroid titers observed in starved pre-CW larvae [68], these data suggest that the upstream developmental and nutritional signaling pathways prior to CW may promote ecdysone biosynthesis by facilitating mitochondrial import of Dib [3].

The nuclear pore complex, composed of nucleoporins (Nups), regulates gene expression by influencing nucleocytoplasmic transport. Nup107 exhibits a distinctive regulatory function during the metamorphic transition in *Drosophila* development. PG-specific depletion of *Nup107* causes larval developmental arrest at the third instar, significantly downregulates the expression of *Spok*, *Phm*, *Dib*, *Sad*, and *Shd*, reduces 20E levels at 120 h after egg laying (AEL), and drastically lowers the EcR nuclear/cytoplasmic ratio. Notably, PG-specific overexpression of *Torso* or *Ras* fully rescues the above phenotypes caused by *Nup107* depletion, indicating that Nup107 is a key regulator of Torso-mediated metamorphic transition, and that Torso-dependent ecdysone synthesis and metamorphosis are modulated by Nup107 levels [119]. Among the upstream signals that induce ecdysone synthesis, the PTTH/Torso pathway is generally considered the primary one [88]. However, recent studies have shown that the epidermal growth factor receptor (Egfr) signaling pathway, activated in an autocrine manner, plays a major role in ecdysone biosynthesis during the mid-to-late third instar by activating the MAPK/ERK pathway in the PG. The PTTH/Torso pathway only acts synergistically to enhance MAPK/ERK activity, thereby accelerating developmental timing, which nicely accounts for why mutants of either *PTTH* or *Torso* only delay, rather than block, the larval-to-pupal transition [120].

## 7. Concluding Remarks

The steroid signaling pathway is evolutionarily conserved between mammals and insects, rendering *Drosophila* an invaluable model for investigating how environmental and intrinsic signals coordinate organismal growth. Larval growth rate and developmental duration act as critical variables shaping the adult body size and proportional morphology, with the precision of pulsed ecdysteroid biosynthesis and downstream signal transduction serving as the central determinant. Over the past decades, multiple core regulators governing ecdysteroid biosynthetic pulses have been identified, including cholesterol availability, transcriptional regulation of the biosynthetic enzymes, and chromatin states. However, recent advances have gradually revealed that these regulatory layers do not operate independently, but rather rely on extensive crosstalk and multi-level integration between external environmental stimuli and internal developmental signals. Despite the identification of a broad spectrum of factors involved in ecdysteroidogenesis, a fundamental question remains, namely how do these factors sense specific intra- and extracellular signals, and through which molecular pathways are they activated or degraded within the appropriate developmental windows to ensure precise hormone pulse output? Addressing this question will lead us toward a fundamentally new understanding of the dynamic regulatory mechanisms that govern steroid hormone biosynthesis.

## Data Availability

No new data were created or analyzed in this study. Data sharing is not applicable to this article.

## References

[1] Herbison A.E. (2016). Control of puberty onset and fertility by gonadotropin-releasing hormone neurons. Nat. Rev. Endocrinol..

[2] Pan X., Connacher R.P., O’Connor M.B. (2021). Control of the insect metamorphic transition by ecdysteroid production and secretion. Curr. Opin. Insect Sci..

[3] Zhang J., Liu S., Li Y., Xu G., Deng H., King-Jones K., Li S. (2025). Nutrient status alters developmental fates via a switch in mitochondrial homeodynamics. Nat. Commun..

[4] Xuan S., Yang T., Ba M., Zhang W., Yang J., Pei X., Qi D., Lu D., Huang S., Li Z. (2025). Chronic restraint stress misaligns corneal clock via β-adrenergic and glucocorticoid signaling, altering epithelial, neural, immune, metabolic states. Investig. Ophthalmol. Vis. Sci..

[5] Hosie A.M., Wilkins M.E., da Silva H.M., Smart T.G. (2006). Endogenous neurosteroids regulate GABAA receptors through two discrete transmembrane sites. Nature.

[6] Kamiyama T., Niwa R. (2022). Transcriptional regulators of ecdysteroid biosynthetic enzymes and their roles in insect development. Front. Physiol..

[7] Jia Q., Li S. (2023). Mmp-induced fat body cell dissociation promotes pupal development and moderately averts pupal diapause by activating lipid metabolism. Proc. Natl. Acad. Sci. USA.

[8] Kang Y., Atif M., Lee Y. (2026). Pleiotropic cholesterol signaling in *Drosophila* and mammalian Systems. Metabolites.

[9] Rewitz K.F., Rybczynski R., Warren J.T., Gilbert L.I. (2006). The Halloween genes code for cytochrome P450 enzymes mediating synthesis of the insect moulting hormone. Biochem. Soc. Trans..

[10] Baptista C., Esteves F., Fallowfield J.A., Kendall T.J., Nelson L.J., Kranendonk M. (2026). Deciphering cytochrome P450 reductase role in MASLD: Molecular mechanisms and pathophysiological implications. Nat. Rev. Gastroenterol. Hepatol..

[11] Yamanaka N., Rewitz K.F., O’Connor M.B. (2013). Ecdysone control of developmental transitions: Lessons from *Drosophila* research. Annu. Rev. Entomol..

[12] Niwa R., Niwa Y.S. (2014). Enzymes for ecdysteroid biosynthesis: Their biological functions in insects and beyond. Biosci. Biotechnol. Biochem..

[13] Yamanaka N., Marqués G., O’Connor M.B. (2015). Vesicle-mediated steroid hormone secretion in *Drosophila melanogaster*. Cell.

[14] Okamoto N., Yamanaka N. (2020). Steroid hormone entry into the brain requires a membrane transporter in *Drosophila*. Curr. Biol..

[15] Warren J.T., Yerushalmi Y., Shimell M.J., O’Connor M.B., Restifo L.L., Gilbert L.I. (2006). Discrete pulses of molting hormone, 20-hydroxyecdysone, during late larval development of *Drosophila melanogaster*: Correlations with changes in gene activity. Dev. Dyn..

[16] Kannangara J.R., Mirth C.K., Warr C.G. (2021). Regulation of ecdysone production in *Drosophila* by neuropeptides and peptide hormones. Open Biol..

[17] Yuan D., Zhou S., Liu S., Li K., Zhao H., Long S., Liu H., Xie Y., Su Y., Yu F. (2020). The AMPK-PP2A axis in insect fat body is activated by 20-hydroxyecdysone to antagonize insulin/IGF signaling and restrict growth rate. Proc. Natl. Acad. Sci. USA.

[18] Knapp E., Sun J. (2017). Steroid signaling in mature follicles is important for *Drosophila* ovulation. Proc. Natl. Acad. Sci. USA.

[19] Strassburger K., Lutz M., Müller S., Teleman A.A. (2021). Ecdysone regulates *Drosophila* wing disc size via a TORC1 dependent mechanism. Nat. Commun..

[20] Parra-Peralbo E., Culi J. (2011). *Drosophila* lipophorin receptors mediate the uptake of neutral lipids in oocytes and imaginal disc cells by an endocytosis-independent mechanism. PLoS Genet..

[21] Palm W., Sampaio J.L., Brankatschk M., Carvalho M., Mahmoud A., Shevchenko A., Eaton S. (2012). Lipoproteins in *Drosophila melanogaster*—assembly, function, and influence on tissue lipid composition. PLoS Genet..

[22] Danielsen E.T., Moeller M.E., Yamanaka N., Ou Q., Laursen J.M., Soenderholm C., Zhuo R., Phelps B., Tang K., Zeng J. (2016). A *Drosophila* genome-wide screen identifies regulators of steroid hormone production and developmental timing. Dev. Cell.

[23] Gilbert L.I. (2004). Halloween genes encode P450 enzymes that mediate steroid hormone biosynthesis in *Drosophila melanogaster*. Mol. Cell. Endocrinol..

[24] Yoshiyama-Yanagawa T., Enya S., Shimada-Niwa Y., Yaguchi S., Haramoto Y., Matsuya T., Shiomi K., Sasakura Y., Takahashi S., Asashima M. (2011). The conserved Rieske oxygenase DAF-36/Neverland is a novel cholesterol-metabolizing enzyme. J. Biol. Chem..

[25] Niwa R., Namiki T., Ito K., Shimada-Niwa Y., Kiuchi M., Kawaoka S., Kayukawa T., Banno Y., Fujimoto Y., Shigenobu S. (2010). Non-molting glossy/shroud encodes a short-chain dehydrogenase/reductase that functions in the ‘Black Box’ of the ecdysteroid biosynthesis pathway. Development.

[26] Ono H., Rewitz K.F., Shinoda T., Itoyama K., Petryk A., Rybczynski R., Jarcho M., Warren J.T., Marqués G., Shimell M.J. (2006). Spook and Spookier code for stage-specific components of the ecdysone biosynthetic pathway in Diptera. Dev. Biol..

[27] Ou Q., Magico A., King-Jones K. (2011). Nuclear receptor DHR4 controls the timing of steroid hormone pulses during *Drosophila* development. PLoS Biol..

[28] Warren J.T., Petryk A., Marqués G., Parvy J.P., Shinoda T., Itoyama K., Kobayashi J., Jarcho M., Li Y., O’Connor M.B. (2004). Phantom encodes the 25-hydroxylase of *Drosophila melanogaster* and *Bombyx mori*: A P450 enzyme critical in ecdysone biosynthesis. Insect Biochem. Mol. Biol..

[29] Chávez V.M., Marqués G., Delbecque J.P., Kobayashi K., Hollingsworth M., Burr J., Natzle J.E., O’Connor M.B. (2000). The *Drosophila* disembodied gene controls late embryonic morphogenesis and codes for a cytochrome P450 enzyme that regulates embryonic ecdysone levels. Development.

[30] Warren J.T., Petryk A., Marques G., Jarcho M., Parvy J.P., Dauphin-Villemant C., O’Connor M.B., Gilbert L.I. (2002). Molecular and biochemical characterization of two P450 enzymes in the ecdysteroidogenic pathway of *Drosophila melanogaster*. Proc. Natl. Acad. Sci. USA.

[31] Petryk A., Warren J.T., Marqués G., Jarcho M.P., Gilbert L.I., Kahler J., Parvy J.P., Li Y., Dauphin-Villemant C., O’Connor M.B. (2003). Shade is the *Drosophila* P450 enzyme that mediates the hydroxylation of ecdysone to the steroid insect molting hormone 20-hydroxyecdysone. Proc. Natl. Acad. Sci. USA.

[32] Enya S., Ameku T., Igarashi F., Iga M., Kataoka H., Shinoda T., Niwa R. (2014). A Halloween gene noppera-bo encodes a glutathione S-transferase essential for ecdysteroid biosynthesis via regulating the behaviour of cholesterol in *Drosophila*. Sci. Rep..

[33] Ebihara K., Niwa R. (2023). Compounds Inhibiting Noppera-bo, a Glutathione S-transferase Involved in Insect Ecdysteroid Biosynthesis: Novel Insect Growth Regulators. Biomolecules.

[34] Šťastná K., Musdal Y., Ismail A., Ebihara K., Niwa R., Mannervik B. (2024). Supreme glutathione-dependent ketosteroid isomerase in the yellow-fever transmitting mosquito *Aedes aegypti*. Biochem. Biophys. Res. Commun..

[35] Schumann I., Kenny N., Hui J., Hering L., Mayer G. (2018). Halloween genes in panarthropods and the evolution of the early moulting pathway in Ecdysozoa. R. Soc. Open Sci..

[36] Wang C.F., Zhang Z., Sun W. (2018). Ecdysone oxidase and 3-dehydroecdysone-3β-reductase contribute to the synthesis of ecdysone during early embryonic development of the silkworm. Int. J. Biol. Sci..

[37] Dermauw W., Van Leeuwen T., Feyereisen R. (2020). Diversity and evolution of the P450 family in arthropods. Insect Biochem. Mol. Biol..

[38] Texada M.J., Koyama T., Rewitz K. (2020). Regulation of body size and growth control. Genetics.

[39] Simon E., Bonche R., Maarouf Y., Nawrot-Esposito M.P., Romero N.M. (2025). Filopodia are essential for steroid release. Nat. Commun..

[40] Wen D., Chen Z., Wen J., Jia Q. (2023). Sterol regulation of development and 20-Hydroxyecdysone biosynthetic and signaling genes in *Drosophila melanogaster*. Cells.

[41] Bradic I., Rewitz K. (2025). Steroid signaling in autophagy. J. Mol. Biol..

[42] Fluegel M.L., Parker T.J., Pallanck L.J. (2006). Mutations of a *Drosophila* NPC1 gene confer sterol and ecdysone metabolic defects. Genetics.

[43] Huang X., Warren J.T., Buchanan J., Gilbert L.I., Scott M.P. (2007). *Drosophila* Niemann-Pick type C-2 genes control sterol homeostasis and steroid biosynthesis: A model of human neurodegenerative disease. Development.

[44] Roth G.E., Gierl M.S., Vollborn L., Meise M., Lintermann R., Korge G. (2004). The *Drosophila* gene Start1: A putative cholesterol transporter and key regulator of ecdysteroid synthesis. Proc. Natl. Acad. Sci. USA.

[45] Zeng J., Kamiyama T., Niwa R., King-Jones K. (2018). The *Drosophila* CCR4-NOT complex is required for cholesterol homeostasis and steroid hormone synthesis. Dev. Biol..

[46] Texada M.J., Malita A., Rewitz K. (2019). Autophagy regulates steroid production by mediating cholesterol trafficking in endocrine cells. Autophagy.

[47] Texada M.J., Malita A., Christensen C.F., Dall K.B., Faergeman N.J., Nagy S., Halberg K.A., Rewitz K. (2019). Autophagy-mediated cholesterol trafficking controls steroid production. Dev. Cell.

[48] Mirth C.K., Riddiford L.M. (2007). Size assessment and growth control: How adult size is determined in insects. Bioessays.

[49] Pan X., Neufeld T.P., O’Connor M.B. (2019). A Tissue- and temporal-specific autophagic switch controls *Drosophila* pre-metamorphic nutritional checkpoints. Curr. Biol..

[50] Xiang Y., Liu Z., Huang X. (2010). br regulates the expression of the ecdysone biosynthesis gene npc1. Dev. Biol..

[51] Zhou X., Zhou B., Truman J.W., Riddiford L.M. (2004). Overexpression of broad: A new insight into its role in the *Drosophila* prothoracic gland cells. J. Exp. Biol..

[52] Horner M.A., Pardee K., Liu S., King-Jones K., Lajoie G., Edwards A., Krause H.M., Thummel C.S. (2009). The *Drosophila* DHR96 nuclear receptor binds cholesterol and regulates cholesterol homeostasis. Genes Dev..

[53] Bujold M., Gopalakrishnan A., Nally E., King-Jones K. (2010). Nuclear receptor DHR96 acts as a sentinel for low cholesterol concentrations in *Drosophila melanogaster*. Mol. Cell. Biol..

[54] Neubueser D., Warren J.T., Gilbert L.I., Cohen S.M. (2005). molting defective is required for ecdysone biosynthesis. Dev. Biol..

[55] Danielsen E.T., Moeller M.E., Dorry E., Komura-Kawa T., Fujimoto Y., Troelsen J.T., Herder R., O’Connor M.B., Niwa R., Rewitz K.F. (2014). Transcriptional control of steroid biosynthesis genes in the *Drosophila* prothoracic gland by ventral veins lacking and knirps. PLoS Genet..

[56] Uryu O., Ou Q., Komura-Kawa T., Kamiyama T., Iga M., Syrzycka M., Hirota K., Kataoka H., Honda B.M., King-Jones K. (2018). Cooperative Control of Ecdysone Biosynthesis in *Drosophila* by Transcription Factors Séance, Ouija Board, and Molting Defective. Genetics.

[57] Kamiyama T., Sun W., Tani N., Nakamura A., Niwa R. (2020). Poly(A) binding protein is required for nuclear localization of the ecdysteroidogenic transcription factor molting defective in the prothoracic gland of *Drosophila melanogaster*. Front. Genet..

[58] Talamillo A., Sánchez J., Cantera R., Pérez C., Martín D., Caminero E., Barrio R. (2008). Smt3 is required for *Drosophila melanogaster* metamorphosis. Development.

[59] Komura-Kawa T., Hirota K., Shimada-Niwa Y., Yamauchi R., Shimell M., Shinoda T., Fukamizu A., O’Connor M.B., Niwa R. (2015). The *Drosophila* zinc finger transcription factor ouija board controls ecdysteroid biosynthesis through specific regulation of spookier. PLoS Genet..

[60] Herold M., Bartkuhn M., Renkawitz R. (2012). CTCF: Insights into insulator function during development. Development.

[61] Kaushal A., Mohana G., Dorier J., Özdemir I., Omer A., Cousin P., Semenova A., Taschner M., Dergai O., Marzetta F. (2021). CTCF loss has limited effects on global genome architecture in *Drosophila* despite critical regulatory functions. Nat. Commun..

[62] Fresán U., Cuartero S., O’Connor M.B., Espinàs M.L. (2015). The insulator protein CTCF regulates *Drosophila* steroidogenesis. Biol. Open.

[63] Zhang T., Song W., Li Z., Qian W., Wei L., Yang Y., Wang W., Zhou X., Meng M., Peng J. (2018). Krüppel homolog 1 represses insect ecdysone biosynthesis by directly inhibiting the transcription of steroidogenic enzymes. Proc. Natl. Acad. Sci. USA.

[64] Liu S., Li K., Gao Y., Liu X., Chen W., Ge W., Feng Q., Palli S.R., Li S. (2018). Antagonistic actions of juvenile hormone and 20-hydroxyecdysone within the ring gland determine developmental transitions in *Drosophila*. Proc. Natl. Acad. Sci. USA.

[65] Nieto M.A. (2002). The snail superfamily of zinc-finger transcription factors. Nat. Rev. Mol. Cell Biol..

[66] Barrallo-Gimeno A., Nieto M.A. (2005). The Snail genes as inducers of cell movement and survival: Implications in development and cancer. Development.

[67] Zeng J., Huynh N., Phelps B., King-Jones K. (2020). Snail synchronizes endocycling in a TOR-dependent manner to coordinate entry and escape from endoreplication pausing during the *Drosophila* critical weight checkpoint. PLoS Biol..

[68] Ohhara Y., Kobayashi S., Yamanaka N. (2017). Nutrient-dependent endocycling in steroidogenic tissue dictates timing of metamorphosis in *Drosophila melanogaster*. PLoS Genet..

[69] Wismar J., Habtemichael N., Warren J.T., Dai J.D., Gilbert L.I., Gateff E. (2000). The mutation without children(rgl) causes ecdysteroid deficiency in third-instar larvae of *Drosophila melanogaster*. Dev. Biol..

[70] Warren J.T., Wismar J., Subrahmanyam B., Gilbert L.I. (2001). Woc (without children) gene control of ecdysone biosynthesis in *Drosophila melanogaster*. Mol. Cell. Endocrinol..

[71] Yoshiyama T., Namiki T., Mita K., Kataoka H., Niwa R. (2006). Neverland is an evolutionally conserved Rieske-domain protein that is essential for ecdysone synthesis and insect growth. Development.

[72] Di Mauro G., Carbonell A., Escudero-Ferruz P., Azorín F. (2020). The zinc-finger proteins WOC and ROW play distinct functions within the HP1c transcription complex. Biochim. Biophys. Acta Gene Regul. Mech..

[73] de Celis J.F., Barrio R. (2009). Regulation and function of Spalt proteins during animal development. Int. J. Dev. Biol..

[74] Ostalé C.M., Pulido D., Vega-Cuesta P., López-Varea A., de Celis J.F. (2024). Developmental analysis of Spalt function in the *Drosophila* prothoracic gland. Development.

[75] Moeller M.E., Danielsen E.T., Herder R., O’Connor M.B., Rewitz K.F. (2013). Dynamic feedback circuits function as a switch for shaping a maturation-inducing steroid pulse in *Drosophila*. Development.

[76] Ou Q., King-Jones K. (2013). What goes up must come down: Transcription factors have their say in making ecdysone pulses. Curr. Top. Dev. Biol..

[77] He Q., Chen J., Chen S., Gao X. (2025). SUMOylation modulates the dual functions of Krüppel homolog 1 in transcriptional regulation of Broad-Complex expression. J. Adv. Res..

[78] Koyama T., Rodrigues M.A., Athanasiadis A., Shingleton A.W., Mirth C.K. (2014). Nutritional control of body size through FoxO-Ultraspiracle mediated ecdysone biosynthesis. eLife.

[79] Jünger M.A., Rintelen F., Stocker H., Wasserman J.D., Végh M., Radimerski T., Greenberg M.E., Hafen E. (2003). The *Drosophila* forkhead transcription factor FOXO mediates the reduction in cell number associated with reduced insulin signaling. J. Biol..

[80] Gao Y., Liu S., Jia Q., Wu L., Yuan D., Li E.Y., Feng Q., Wang G., Palli S.R., Wang J. (2022). Juvenile hormone membrane signaling phosphorylates USP and thus potentiates 20-hydroxyecdysone action in *Drosophila*. Sci. Bull..

[81] Parvy J.P., Wang P., Garrido D., Maria A., Blais C., Poidevin M., Montagne J. (2014). Forward and feedback regulation of cyclic steroid production in *Drosophila melanogaster*. Development.

[82] Niwa Y.S., Niwa R. (2016). Transcriptional regulation of insect steroid hormone biosynthesis and its role in controlling timing of molting and metamorphosis. Dev. Growth Differ..

[83] Parvy J.P., Blais C., Bernard F., Warren J.T., Petryk A., Gilbert L.I., O’Connor M.B., Dauphin-Villemant C. (2005). A role for betaFTZ-F1 in regulating ecdysteroid titers during post-embryonic development in *Drosophila melanogaster*. Dev. Biol..

[84] Borsos B.N., Pankotai T., Kovács D., Popescu C., Páhi Z., Boros I.M. (2015). Acetylations of Ftz-F1 and histone H4K5 are required for the fine-tuning of ecdysone biosynthesis during *Drosophila metamorphosis*. Dev. Biol..

[85] Bialecki M., Shilton A., Fichtenberg C., Segraves W.A., Thummel C.S. (2002). Loss of the ecdysteroid-inducible E75A orphan nuclear receptor uncouples molting from metamorphosis in *Drosophila*. Dev. Cell.

[86] Cáceres L., Necakov A.S., Schwartz C., Kimber S., Roberts I.J., Krause H.M. (2011). Nitric oxide coordinates metabolism, growth, and development via the nuclear receptor E75. Genes Dev..

[87] King-Jones K., Charles J.P., Lam G., Thummel C.S. (2005). The ecdysone-induced DHR4 orphan nuclear receptor coordinates growth and maturation in *Drosophila*. Cell.

[88] McBrayer Z., Ono H., Shimell M., Parvy J.P., Beckstead R.B., Warren J.T., Thummel C.S., Dauphin-Villemant C., Gilbert L.I., O’Connor M.B. (2007). Prothoracicotropic hormone regulates developmental timing and body size in *Drosophila*. Dev. Cell.

[89] Rewitz K.F., Yamanaka N., Gilbert L.I., O’Connor M.B. (2009). The insect neuropeptide PTTH activates receptor tyrosine kinase torso to initiate metamorphosis. Science.

[90] Jaeger J. (2011). The gap gene network. Cell. Mol. Life Sci..

[91] Deng H., Kerppola T.K. (2013). Regulation of *Drosophila* metamorphosis by xenobiotic response regulators. PLoS Genet..

[92] Pitoniak A., Bohmann D. (2015). Mechanisms and functions of Nrf2 signaling in *Drosophila*. Free Radic. Biol. Med..

[93] Ulasov A.V., Rosenkranz A.A., Georgiev G.P., Sobolev A.S. (2022). Nrf2/Keap1/ARE signaling: Towards specific regulation. Life Sci..

[94] Carlson J., Price L., Cook I., Deng H. (2022). *Drosophila* Keap1 xenobiotic response factor regulates developmental transcription through binding to chromatin. Dev. Biol..

[95] Saha T.T., Shin S.W., Dou W., Roy S., Zhao B., Hou Y., Wang X.L., Zou Z., Girke T., Raikhel A.S. (2016). Hairy and Groucho mediate the action of juvenile hormone receptor Methoprene-tolerant in gene repression. Proc. Natl. Acad. Sci. USA.

[96] Saha T.T., Roy S., Pei G., Dou W., Zou Z., Raikhel A.S. (2019). Synergistic action of the transcription factors Krüppel homolog 1 and Hairy in juvenile hormone/Methoprene-tolerant-mediated gene-repression in the mosquito *Aedes aegypti*. PLoS Genet..

[97] Yang Y., Zhao T., Li Z., Qian W., Peng J., Wei L., Yuan D., Li Y., Xia Q., Cheng D. (2021). Histone H3K27 methylation-mediated repression of Hairy regulates insect developmental transition by modulating ecdysone biosynthesis. Proc. Natl. Acad. Sci. USA.

[98] Yuan D., Zhang X., Yang Y., Wei L., Li H., Zhao T., Guo M., Li Z., Huang Z., Wang M. (2024). Schlank orchestrates insect developmental transition by switching H3K27 acetylation to trimethylation in the prothoracic gland. Proc. Natl. Acad. Sci. USA.

[99] Cheng C., Ko A., Chaieb L., Koyama T., Sarwar P., Mirth C.K., Smith W.A., Suzuki Y. (2014). The POU factor ventral veins lacking/Drifter directs the timing of metamorphosis through ecdysteroid and juvenile hormone signaling. PLoS Genet..

[100] Meng M., Cheng D.J., Peng J., Qian W.L., Li J.R., Dai D.D., Zhang T.L., Xia Q.Y. (2015). The homeodomain transcription factors antennapedia and POU-M2 regulate the transcription of the steroidogenic enzyme gene Phantom in the silkworm. J. Biol. Chem..

[101] Sarwar P.F., McDonald I.R., Wang V.R., Suzuki Y. (2020). The POU factor Ventral veins lacking regulates ecdysone and juvenile hormone biosynthesis during development and reproduction of the milkweed bug, *Oncopeltus fasciatus*. Dev. Biol..

[102] Hellen C.U.T. (2018). Translation termination and ribosome recycling in eukaryotes. Cold Spring Harb. Perspect. Biol..

[103] Zhao Y., Lindberg B.G., Esfahani S.S., Tang X., Piazza S., Engström Y. (2021). Stop codon readthrough alters the activity of a POU/Oct transcription factor during *Drosophila* development. BMC Biol..

[104] Wangen J.R., Green R. (2020). Stop codon context influences genome-wide stimulation of termination codon readthrough by aminoglycosides. eLife.

[105] Liu W., Yan M., King-Jones K. (2024). Soul is a master control gene governing the development of the *Drosophila* prothoracic gland. Proc. Natl. Acad. Sci. USA.

[106] Ou Q., Zeng J., Yamanaka N., Brakken-Thal C., O’Connor M.B., King-Jones K. (2016). The insect prothoracic gland as a model for steroid hormone biosynthesis and regulation. Cell Rep..

[107] Pankotai T., Popescu C., Martín D., Grau B., Zsindely N., Bodai L., Tora L., Ferrús A., Boros I. (2010). Genes of the ecdysone biosynthesis pathway are regulated by the dATAC histone acetyltransferase complex in *Drosophila*. Mol. Cell. Biol..

[108] Ohhara Y., Nakamura A., Kato Y., Yamakawa-Kobayashi K. (2019). Chaperonin TRiC/CCT supports mitotic exit and entry into endocycle in *Drosophila*. PLoS Genet..

[109] Drelon C., Rogers M.F., Belalcazar H.M., Secombe J. (2019). The histone demethylase KDM5 controls developmental timing in *Drosophila* by promoting prothoracic gland endocycles. Development.

[110] Lu B.Y., Emtage P.C., Duyf B.J., Hilliker A.J., Eissenberg J.C. (2000). Heterochromatin protein 1 is required for the normal expression of two heterochromatin genes in *Drosophila*. Genetics.

[111] Yasuhara J.C., Wakimoto B.T. (2006). Oxymoron no more: The expanding world of heterochromatic genes. Trends Genet..

[112] Ohhara Y., Kato Y., Kamiyama T., Yamakawa-Kobayashi K. (2022). Su(var)2-10- and Su(var)205-dependent upregulation of the heterochromatic gene neverland is required for developmental transition in *Drosophila*. Genetics.

[113] Ebert A., Schotta G., Lein S., Kubicek S., Krauss V., Jenuwein T., Reuter G. (2004). Su(var) genes regulate the balance between euchromatin and heterochromatin in *Drosophila*. Genes Dev..

[114] Elgin S.C., Reuter G. (2013). Position-effect variegation, heterochromatin formation, and gene silencing in *Drosophila*. Cold Spring Harb. Perspect. Biol..

[115] Enya S., Yamamoto C., Mizuno H., Esaki T., Lin H.K., Iga M., Morohashi K., Hirano Y., Kataoka H., Masujima T. (2017). Dual roles of glutathione in ecdysone biosynthesis and antioxidant function during larval development in *Drosophila*. Genetics.

[116] Palandri A., L’hôte D., Cohen-Tannoudji J., Tricoire H., Monnier V. (2015). Frataxin inactivation leads to steroid deficiency in flies and human ovarian cells. Hum. Mol. Genet..

[117] Uhrigshardt H., Rouault T.A., Missirlis F. (2013). Insertion mutants in *Drosophila melanogaster* Hsc20 halt larval growth and lead to reduced iron-sulfur cluster enzyme activities and impaired iron homeostasis. J. Biol. Inorg. Chem..

[118] Llorens J.V., Metzendorf C., Missirlis F., Lind M.I. (2015). Mitochondrial iron supply is required for the developmental pulse of ecdysone biosynthesis that initiates metamorphosis in *Drosophila melanogaster*. J. Biol. Inorg. Chem..

[119] Kawadkar J., Joshi P.A., Mishra R.K. (2026). Nup107 is a crucial regulator of torso-mediated metamorphic transition in *Drosophila melanogaster*. eLife.

[120] Cruz J., Martín D., Franch-Marro X. (2020). Egfr signaling is a major regulator of ecdysone biosynthesis in the *Drosophila* prothoracic gland. Curr. Biol..

